# A nerve-goblet cell association promotes allergic conjunctivitis through rapid antigen passage

**DOI:** 10.1172/jci.insight.168596

**Published:** 2023-11-08

**Authors:** Meiko Kimura, Tomoaki Ando, Yasuharu Kume, Saaya Fukase, Moe Matsuzawa, Kosuke Kashiwagi, Kumi Izawa, Ayako Kaitani, Nobuhiro Nakano, Keiko Maeda, Hideoki Ogawa, Ko Okumura, Shintaro Nakao, Akira Murakami, Nobuyuki Ebihara, Jiro Kitaura

**Affiliations:** 1 Atopy Research Center, Juntendo University Graduate School of Medicine, Bunkyo-ku, Tokyo, Japan.; 2 Department of Ophthalmology, Juntendo University Urayasu Hospital, Urayasu, Chiba, Japan.; 3 Department of Ophthalmology,; 4 Department of Pediatrics and Adolescent Medicine, and; 5 Department of Science of Allergy and Inflammation, Juntendo University Graduate School of Medicine, Bunkyo-ku, Tokyo, Japan.

**Keywords:** Immunology, Ophthalmology, Allergy

## Abstract

The penetration of allergens through the epithelial layer is the initial step in the development of allergic conjunctivitis. Although pollinosis patients manifest symptoms within minutes after pollen exposure, the mechanisms of the rapid transport of the allergens remain unclear. In the present study, we found that the instillation of pollen shells rapidly induces a large number of goblet cell–associated antigen passages (GAPs) in the conjunctiva. Antigen acquisition by stromal cells, including macrophages and CD11b^+^ dendritic cells, correlated with surface GAP formation. Furthermore, a substantial amount of antigen was transported to the stroma during the first 10 minutes of pollen exposure, which was sufficient for the full induction of an allergic conjunctivitis mouse model. This inducible, rapid GAP formation and antigen acquisition were suppressed by topical lidocaine or trigeminal nerve ablation, indicating that the sensory nervous system plays an essential role. Interestingly, pollen shell–stimulated GAP formation was not suppressed by topical atropine, suggesting that the conjunctival GAPs and intestinal GAPs are differentially regulated. These results identify pollen shell–induced GAP as a therapeutic target for allergic conjunctivitis.

## Graphical abstract

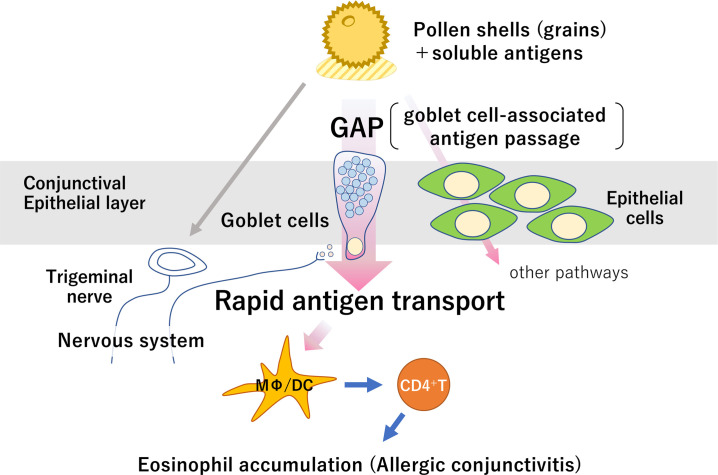

## Introduction

Goblet cells are specialized epithelial cell types found in wet body surfaces. The primary role for goblet cells is mucus secretion, which is essential for the maintenance of the mucosal tissues, including the intestinal, airway, and ocular surfaces. For instance, the loss of Muc2, an intestinal gel-forming mucin, leads to the development of spontaneous colitis and ultimately to carcinogenesis (1, 2). The mucus granules of conjunctival goblet cells contain mainly Muc5ac gel-forming mucin. These mucus granules are exocytosed, regularly and upon stimulation (3). The released mucins absorb water to form a gel-like structure and contribute to hydration, lubrication, and removal of debris on ocular surfaces (4, 5).

In addition to the functions attributable to the physical properties of mucus, goblet cells have immunoregulatory roles. In the intestine, the glycans associated with the Muc2 molecule assemble a galectin-3–Dectin-1–FcγRIIB receptor complex on dendritic cells (DCs) and skew the cytokine profiles of DCs into tolerogenic ones (6). In the conjunctiva, retinoic acid produced by conjunctival goblet cells reduces IL-12 production from DCs, and the loss of goblet cells attenuates the tolerogenic response in the conjunctiva to suppress the Th1-type delayed hypersensitivity reaction in the skin (7). In addition, cultured conjunctival goblet cells produce soluble factors, including TGF-β2, which alter the DC phenotype into a tolerogenic type (8). Furthermore, goblet cells of both the intestine and conjunctiva form a goblet cell–associated antigen passage (GAP), which transfers luminal antigens to DCs in the stroma (9, 10). Goblet cells are associated with the maintenance of tolerance to type 1/17 inflammation in both tissues (7, 9, 11). However, the transgenic expression of SAM-pointed domain–containing ETS-like factor (Spdef) in airway epithelial cells induced goblet cell metaplasia and spontaneous type 2 pulmonary inflammation. Conversely, the deletion of Spdef abrogated the asthmatic responses in a house dust mite–induced airway inflammation model (12). In addition, cultured human airway goblet cells express type 2 inflammatory cytokines (13). These observations suggest that goblet cells may promote inflammation in the context of type 2 allergic reactions (4). However, the roles of GAPs in the development of allergic inflammation remain unclear.

The prevalence of allergic conjunctival diseases has increased in recent years to up to 50%, depending on the country (14–16). These conditions have several forms, ranging from mild but recurrent or persistent seasonal and perennial allergic conjunctivitis (SAC and PAC, respectively) to severe forms such as vernal keratoconjunctivitis (VKC) and atopic keratoconjunctivitis (AKC), which potentially result in corneal damage and vision loss (17–22). Allergic conjunctival diseases often accompany other allergic diseases, including allergic rhinitis, asthma (23), atopic eczema (17, 24), and food allergy (18, 25). However, isolated ocular symptoms have also been observed in approximately 6% of the general population (15) and in up to 60% of ocular allergy patients consulting ophthalmologists (26). Patients without suspected allergen-specific IgE in the circulation have also been reported, raising the concept of local ocular allergy and local sensitization in VKC and allergic conjunctivitis (27–30).

To better understand the mechanisms underlying ocular sensitization, a local sensitization model of allergic conjunctivitis was recently established (31). Interestingly, repeated applications of ragweed (RW) pollen, but not its extract or shell alone, provoked a marked increase in eosinophils in the conjunctiva. A combination of pollen extracts and shells restored the eosinophilic inflammation, suggesting that the pollen shells and soluble factors play nonredundant roles (31). The conjunctival eosinophilia was dependent on CD4^+^ T cells and the IL-33 receptor ST2, and the numbers of CD4^+^ST2^+^ T cells were highly correlated with those of infiltrating eosinophils in the conjunctiva. Furthermore, one of the roles of the shells was to stimulate IL-33 release from the conjunctival epithelial cells (31). However, the roles of the soluble factors, and the pathways through which protein antigens are acquired during the development of allergic conjunctivitis, remain obscure.

In the present study, we initially examined whether any specific properties of soluble factors were required for local sensitization. Surprisingly, ovalbumin (OVA), an inert protein antigen, was sufficient to induce eosinophilic conjunctivitis when combined with pollen shells. The successful induction of allergic conjunctivitis allowed us to track the pathways of antigen acquisition through the conjunctiva using fluorescently labeled OVA. Using this strategy, we have revealed that the goblet cells play an essential role in rapidly acquiring protein antigen by forming GAPs. These GAPs were distinct from the previously reported intestinal GAPs, in that the regulatory pathways do not use muscarinic acetylcholine receptors (mAchRs). We further investigated the significance of the GAP formation and its regulatory mechanisms in the development of allergic conjunctivitis.

## Results

### OVA elicits eosinophilic inflammation when combined with pollen shells.

**Figure 1:**
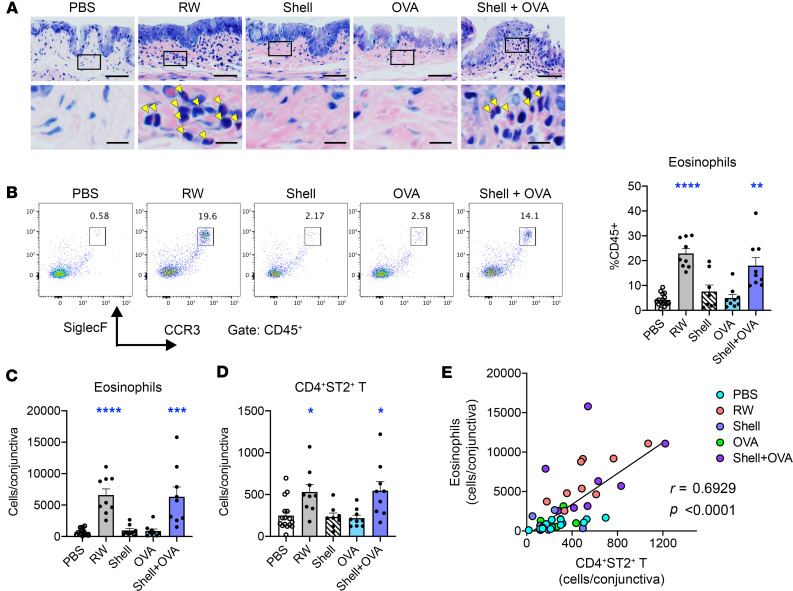
Repeated topical application of OVA combined with RW pollen shells elicits eosinophilic conjunctivitis. (**A**) Giemsa staining of the conjunctival tissue at ×200 magnification. Scale bars: 50 μm (lower magnification) and 10 μm (insets). Arrowheads denote eosinophils. (**B**–**D**) Conjunctival eosinophil populations among CD45^+^ cells (**B** and **C**) and cell numbers of indicated populations in the conjunctiva (**D**). Pooled results of 2 independent experiments (*n* = 8–18). Data are shown as mean ± SEM. *P < 0.05, **P < 0.01, ***P < 0.001, ****P < 0.0001 by 2-tailed Kruskal-Wallis test with Dunn’s multiple-comparison test. (**E**) Pearson’s correlation between CD4^+^ST2^+^ T cell and eosinophil numbers in the conjunctiva. The B6 mouse strain was used for all experiments.

We previously reported that both pollen shells and soluble factors are required for the development of allergic conjunctivitis (31). Although pollen shells play a role in the mobilization of IL-33 from conjunctival epithelial cells, the role of soluble factors remains obscure. To elucidate whether any of the properties of pollen soluble factors, such as protease activity, are required for the development of allergic conjunctivitis, we tested whether an inert protein, OVA, can induce allergic conjunctivitis when combined with pollen shells. Repeated topical applications of pollen shells or OVA to the conjunctiva did not induce eosinophilic conjunctivitis (Figure 1, A–D). However, a combination of the 2 induced the accumulation of eosinophils to levels similar to those induced by RW pollen administration (Figure 1, A–D). Consistently with our previous report, the number of CD4^+^ST2^+^ T cells was correlated with that of eosinophils, suggesting that the type 2 inflammation was induced by the combination of OVA and pollen shells (Figure 1, D and E). A previous report suggested a major role of T cells and a minor role of IgE in late-phase eosinophil accumulation (32). Therefore, we examined whether loss of the high-affinity IgE receptor, FcεRI, affects eosinophil accumulation in our model (Supplemental Figure 1A; supplemental material available online with this article; https://doi.org/10.1172/jci.insight.168596DS1). Interestingly, FcεRI-deficient mice did not show a decrease in eosinophils, suggesting that the eosinophilic late-phase reactions are largely mediated by non-IgE reactions in our model (Supplemental Figure 1B). To further elucidate the pollen shell contribution in the elicitation phase, the mice sensitized with topical administration of OVA and RW pollen shells for 3 weeks received the last challenge with PBS, OVA alone, or OVA and RW pollen shells (Supplemental Figure 1C). Some eosinophilic inflammation was readily observed after 3 weeks of allergen exposure, even when PBS was used for the last challenge (Supplemental Figure 1D). Compared with this as a control, the last challenge with OVA increased eosinophil numbers only marginally (but not significantly), while OVA and RW pollen shell significantly increased the eosinophil numbers (Supplemental Figure 1D). These results indicate that the RW pollen shells are required for sensitization and enhances the elicitation-phase reaction.

### Major populations taking up antigen include macrophages, CD11b^+^ DCs, stromal cells, and epithelial cells.

**Figure 2:**
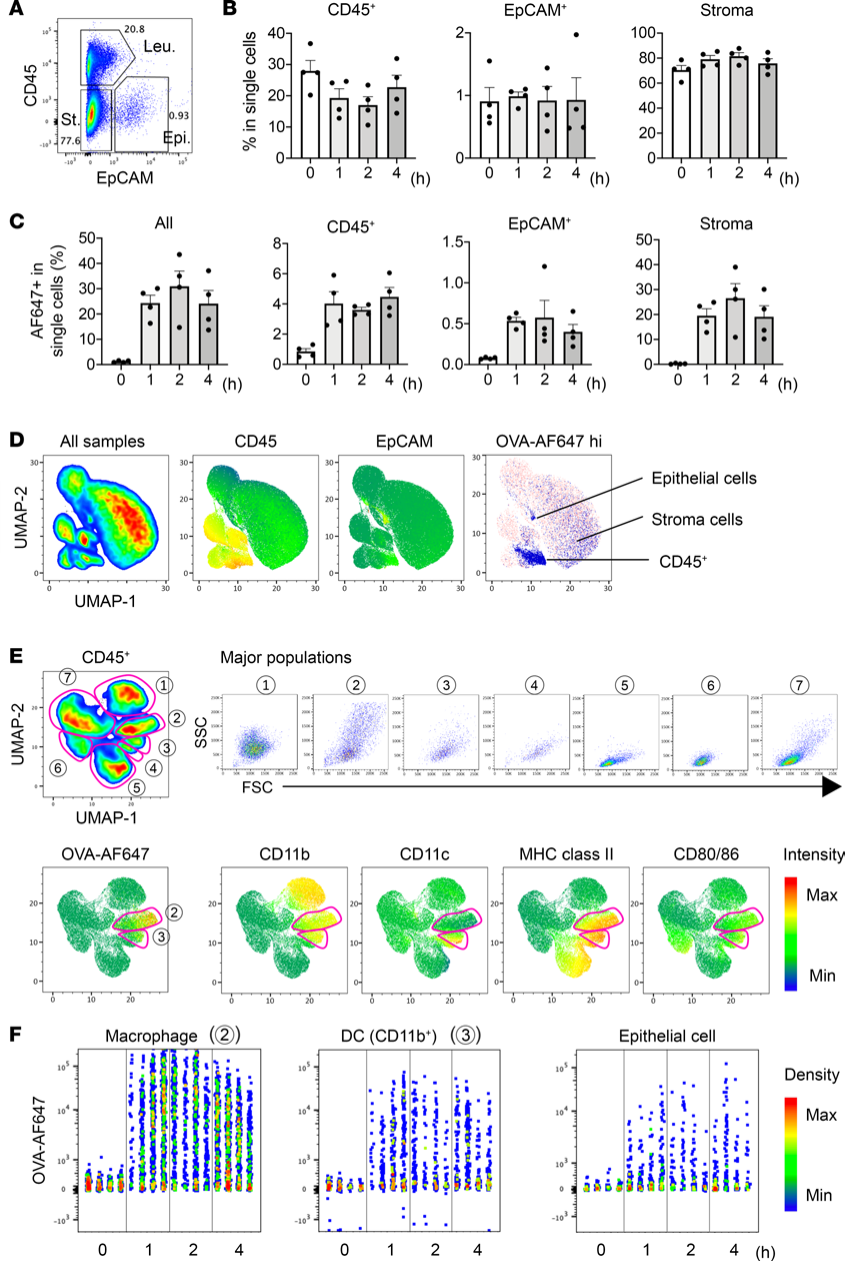
Kinetics and cell populations for antigen uptake. (**A**) Frequencies of the pooled dispersed conjunctival cell populations. Leu, leukocytes; Epi, epithelial cells; St, stromal cells. (**B** and **C**) Kinetics of the frequencies of indicated cell populations (**B**) and those of indicated cell populations that are OVA-AF647 positive (**C**) (*n* = 4, each time point). Data are shown as mean ± SEM (**B** and **C**). (**D**) Unsupervised clustering of the conjunctival cells. The expression levels of CD45 and EpCAM are shown in heatmaps. OVA-AF647–high cells are shown in blue. (**E**) Unsupervised clustering of the CD45^+^ cells revealed 7 distinct populations with differential forward- and side-scatter distributions. The expression levels of the indicated markers are shown in heatmaps. (**F**) Kinetics of OVA-AF647 uptake by the indicated cell populations (*n* = 4, each time point). B6 mice were used for all experiments.

As OVA was sufficient as a soluble antigen for the development of eosinophilic conjunctivitis when combined with pollen shells, Alexa Fluor 647–labeled OVA (OVA-AF647) was used to track the applied antigen. The cells that dispersed from the conjunctiva contained CD45^+^ leukocytes, EpCAM^+^ epithelial cells, and double-negative stromal cells (Figure 2A); the cell composition was stable for up to 4 hours after the administration of pollen shells and OVA-AF647 (Figure 2B). Interestingly, the antigen uptake by these 3 populations plateaued as early as 1 hour (Figure 2C). To identify the cells that were acquiring antigens, we performed an unsupervised clustering of the cell populations using cell surface markers (Figure 2, D and E). Most of the OVA-AF647–high populations were found in the subpopulations of CD45^+^ cells, although some epithelial and stromal cells also internalized antigens (Figure 2D). The CD45^+^ cells were subdivided into 7 major populations, of which the macrophages and CD11b^+^ DCs were OVA-AF647 positive (Figure 2E). The kinetics of OVA-AF647 uptake were confirmed in the 2 CD45^+^ cell populations and in epithelial cells (Figure 2F). These results indicate that protein antigens administered together with pollen shells are acquired through the conjunctiva by various cell types, including macrophages and CD11b^+^ DCs, within 1 hour.

### Pollen shells promote GAP formation and antigen uptake in the conjunctiva.

**Figure 3:**
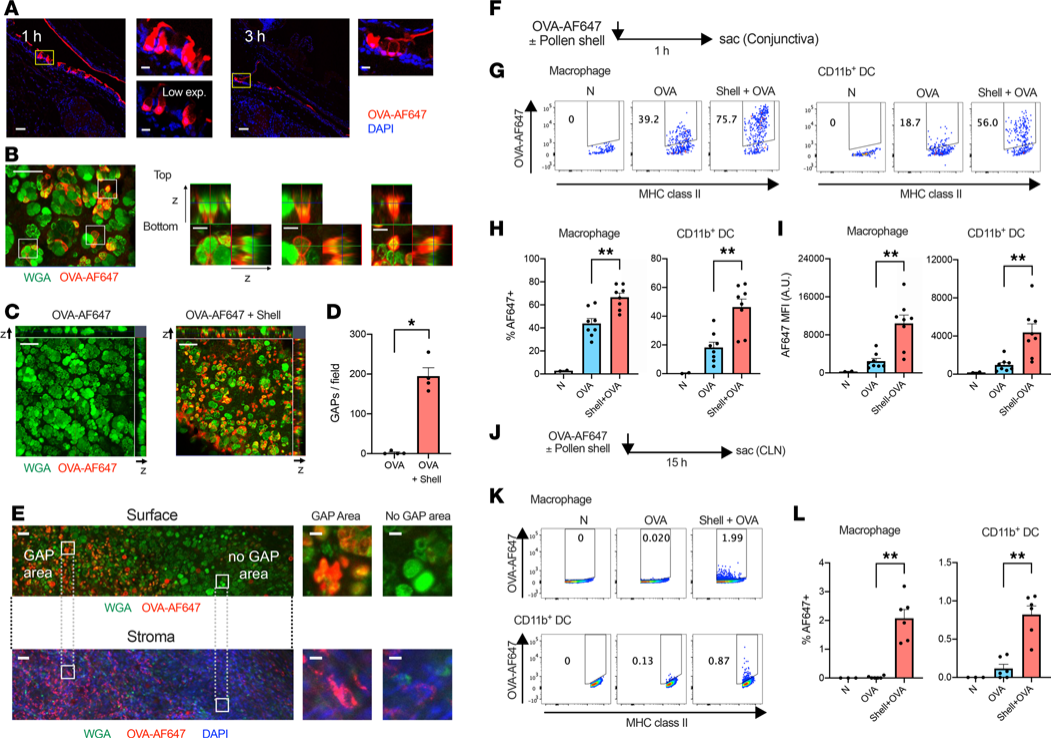
RW pollen shells promote GAP formation and antigen uptake through the conjunctiva. (**A**) Localization of OVA-AF647 in the conjunctiva at the indicated time periods after topical instillation of RW pollen shells and OVA-AF647. (**B**) Representative morphology of GAPs in the conjunctiva. (**C** and **D**) GAP formation in the conjunctiva 5 minutes after instillation of the indicated formula. Representative images (**C**) and quantitation (**D**) (*n* = 4, each condition). *P < 0.05 by 2-tailed Mann-Whitney test. (**E**) Representative images of the conjunctival surface with the corresponding stroma below. Scale bars: 50 μm (lower magnification) and 10 μm (insets) (**A**–**C** and **E**). (**F**) Diagram for OVA-AF647 uptake experiment. Naive mice were challenged once, and the antigen uptake was evaluated. (**G**–**I**) Frequencies of OVA-AF647^+^ cells and mean fluorescence intensity (MFI) in the indicated cell populations (*n* = 2–8). A.U., arbitrary units; N, nontreated. The nontreated samples were used for setting the positive gate and were excluded from the statistical analysis. **P < 0.01 by 2-tailed Student’s *t* test with Welch’s correction. (**J**) Diagram of the antigen transport experiment. CLN, cervical lymph nodes. (**K** and **L**) Frequencies of OVA-AF647^+^ cells in the indicated populations in the cervical lymph nodes (*n* = 3–6). B6 mice were used for all experiments. Data are shown as mean ± SEM (**D**, **H**, **I**, and **L**).

To further clarify the distribution of antigens in the conjunctiva, tissue sections were analyzed. Interestingly, the brightest cells that internalized OVA-AF647 had a goblet cell–like morphology, suggesting that the GAPs were formed after the administration of antigen and pollen shells (Figure 3A). Whole-mount immunostaining using the tissue-clearing technique (33) revealed that the cells internalizing large amounts of OVA-AF647 were wheat germ agglutinin–positive (WGA-positive) goblet cells (Figure 3B). The formation of these GAPs was extremely fast, as they were observed in the tissues that were fixed in situ 5 minutes after the administration of OVA and pollen shells (Figure 3, B and C). The GAPs had various WGA-positive mucus contents, suggesting that complete release of mucus content is not a prerequisite for GAP formation in conjunctival goblet cells (Figure 3B). Intriguingly, the GAP formation was almost completely dependent on the exposure to pollen shells (Figure 3, C and D). Furthermore, the antigen uptake by cells in the stroma was correlated with surface GAP formation (Figure 3E and Supplemental Figure 2). Accordingly, the antigen uptake (Figure 3, F–I) and trafficking to the draining lymph nodes (Figure 3, J–L) increased after exposure to pollen shells. These results indicated that pollen shells stimulated the GAP formation and promoted the antigen uptake and migration of antigen-presenting cells.

### Early antigen passage is essential for antigen uptake and the development of allergic conjunctivitis.

**Figure 4:**
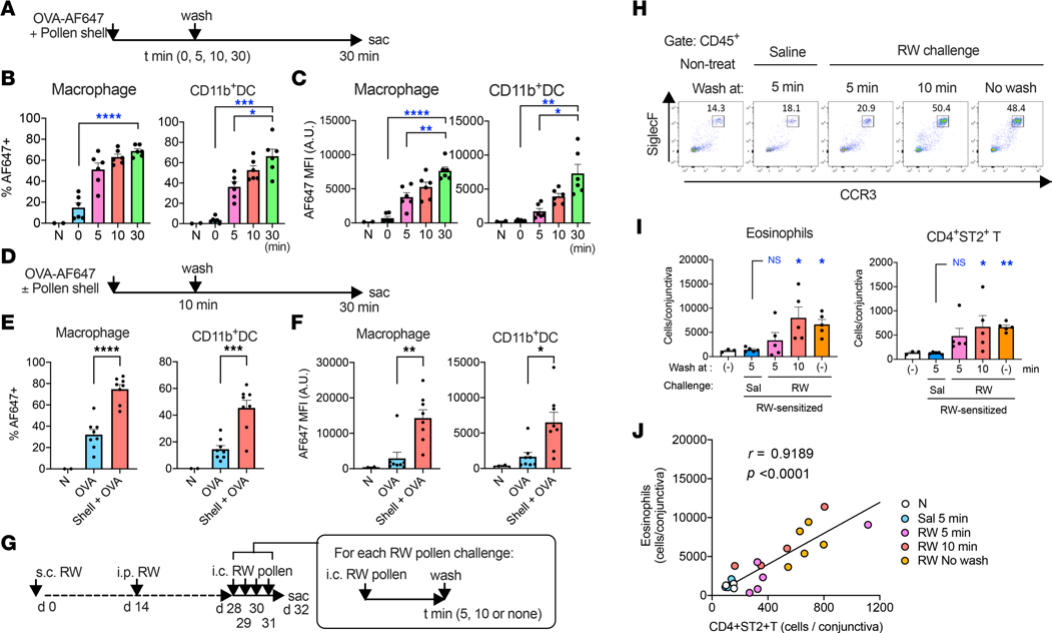
Early antigen passage is essential for the antigen uptake and the development of allergic conjunctivitis. (**A**) Experimental diagram for panels **B** and **C**. (**B** and **C**) The frequencies of OVA-AF647^+^ cells (**B**) and the mean fluorescence intensity (MFI) (**C**) of the indicated cell populations in the conjunctiva that was exposed to OVA-AF647 with RW pollen shells for the indicated periods of time (*n* = 2–6). A.U., arbitrary units; N, nontreated. The nontreated samples were used for setting the positive gate and were excluded from the statistical analysis. *P < 0.05; **P < 0.01; ***P < 0.001; ****P < 0.0001 by 2-tailed Brown-Forsythe and Welch’s ANOVA test with Dunnett’s T3 multiple-comparison test against the 30-minute exposure. (**D**) Experimental diagram for panels **E** and **F**. Naive mice were challenged once, and the antigen uptake was evaluated. (**E** and **F**) The antigen uptake by indicated cell types after instillation of the indicated formula (*n* = 2–8). *P < 0.05, **P < 0.01, ***P < 0.001, ****P < 0.0001 by 2-tailed Student’s *t* test with Welch’s correction. (**G**) Experimental diagram of the systemically sensitized model of allergic conjunctivitis with eye wash at the indicated time points after instillation of pollen suspension. (**H**) Representative gating of eosinophil populations among CD45^+^ cells. (**I** and **J**) Cell numbers of indicated populations (**I**) and their correlation (**J**) (*n* = 3–5). Sal, saline. In **I**, (–) indicates no treatment. Data are shown as mean ± SEM (**B**, **C**, **E**, **F**, and **I**). *P < 0.05, **P < 0.01 by Kruskal-Wallis test with Dunn’s multiple-comparison test against saline-challenged mice. B6 mice were used for **A**–**F** and BALB/c mice were used for **G**–**J**.

The rapid formation of GAPs led us to examine the short-term kinetics of the required antigen exposure in the conjunctiva. After administration of OVA-AF647 and pollen shells, the mouse conjunctiva was washed at a certain time point, and the mouse was rested thereafter until 30 minutes after the challenge (Figure 4A). A 10-minute exposure to allergens was sufficient for the uptake of more than half of the antigens that were acquired through full 30-minute exposure in both macrophages and CD11b^+^ DCs, indicating that at least half of the antigen passage through the conjunctival surface occurred within 10 minutes (Figure 4, B and C). Furthermore, most of the early antigen passage was dependent on the exposure to pollen shells, which coincided with GAP formation (Figure 4, D–F). To assess whether this early antigen passage is required for the development of allergic conjunctivitis, we introduced wash procedures to a mouse model of allergic conjunctivitis. To minimize the effect of mechanical stimuli introduced by the wash procedures, we employed a different model of allergic conjunctivitis, where only 4 final challenges were performed after systemic sensitization (Figure 4G). Because this model requires the BALB/c strain, we tested whether pollen shells promote GAP formation also in BALB/c mice. Consistent with the above results observed in C57BL/6J (B6) mice, GAP development in BALB/c mice was dependent on the pollen shell stimuli, regardless of the systemic sensitization profile (Supplemental Figure 3, A–D). In addition, the early antigen passage through the conjunctival surface was largely dependent on the pollen shells (Supplemental Figure 3, E–G). These results suggest that the pollen shell–induced GAP formation and the early antigen influx is a mouse strain–independent phenomenon. We therefore performed wash procedures during the elicitation phase of the systemically sensitized model of allergic conjunctivitis (Figure 4G). In the elicitation phase, the mouse conjunctiva was exposed to the pollen suspension for a limited time (with wash) or left unwashed once a day for 4 consecutive days. Twenty-four hours after the last challenge, the allergic responses were assessed using flow cytometry (Figure 4G). Interestingly, the levels of eosinophil accumulation in the mice that underwent wash at 10 minutes were similar to those of unwashed mice, suggesting that the 10-minute exposure to particulate pollen allergen was sufficient for full induction of the allergic conjunctivitis (Figure 4, H and I). Importantly, the number of eosinophils correlated well with that of ST2^+^CD4^+^ T cells (Figure 4, I and J). These results indicate that the magnitude of type 2 inflammation is dependent on the early antigen passage through the conjunctiva, presumably through the rapid formation of GAPs.

### Piezo1, IL-33 receptor ST2, H1 histamine receptors, and pH do not affect GAP formation.

To elucidate the underlying mechanisms through which the pollen shells trigger the formation of GAPs, we investigated whether goblet cell–intrinsic and/or paracrine mechanisms are involved. The mechanosensitive receptor Piezo1 has been implicated in the induction of epithelial cell stretch reactions. Therefore, we examined whether cell stretch triggers GAP formation through Piezo1 stimulation. To this end, we administered gadolinium chloride [Gd(III)Cl_3_], which inhibits Ca^2+^ influx through several receptors, including Piezo1 (34) (Supplemental Figure 4A). However, it had no inhibitory effect on GAP formation (Supplemental Figure 4, B and C). Consistently with this, antigen uptake was not altered in Gd(III)-administered mice (Supplemental Figure 4, D–F). These results suggest that Piezo1 does not play a role in detecting particulate antigens.

Other possible mechanisms regulating antigen uptake include downstream signaling of IL-33 released from pollen shell–stimulated epithelial cells (Supplemental Figure 5A). The activation of myeloid differentiation primary response 88 (Myd88) suppresses GAP formation in the colon (35). As IL-33 also signals through the Myd88 adaptor molecule via the IL-33 receptor ST2, it is possible that IL-33 suppresses GAP formation. However, GAP formation was not affected by the loss of IL-33 receptor ST2 (Supplemental Figure 5, B and C). Since IL-33 release occurs within 1 hour after particle exposure (31), the final antigen uptake of the antigen-presenting cells was evaluated after 2 hours (Supplemental Figure 5D). Consistently, antigen uptake was unaltered in ST2-knockout mice (Supplemental Figure 5, E and F). These results indicate that IL-33 signaling was not responsible for the induction of antigen passage through the conjunctiva or the antigen uptake thereafter.

In addition to these receptors, we evaluated the contribution of the H1 histamine receptor to pollen shell–induced GAP formation (Supplemental Figure 6). A commercially available inverse agonist of the H1 histamine receptor, epinastine (36), was instilled to mouse eyes before and during the OVA-AF647 and pollen shell challenges (Supplemental Figure 6A). However, epinastine did not affect GAP formation, suggesting that prophylactic antihistamine does not ameliorate allergic conjunctivitis through GAP suppression (Supplemental Figure 6, B and C).

We further examined the effects of pH, since the pHs of some ophthalmic solutions are not exactly neutral. We found that pollen shell–stimulated GAP formation was unaffected by pH ranging from pH 6 to pH 8 (Supplemental Figure 7). The results suggest that most of the close-to-neutral ophthalmic formulations may not affect GAP formation through their pH.

### Topical lidocaine reduces GAP formation and early antigen passage.

**Figure 5:**
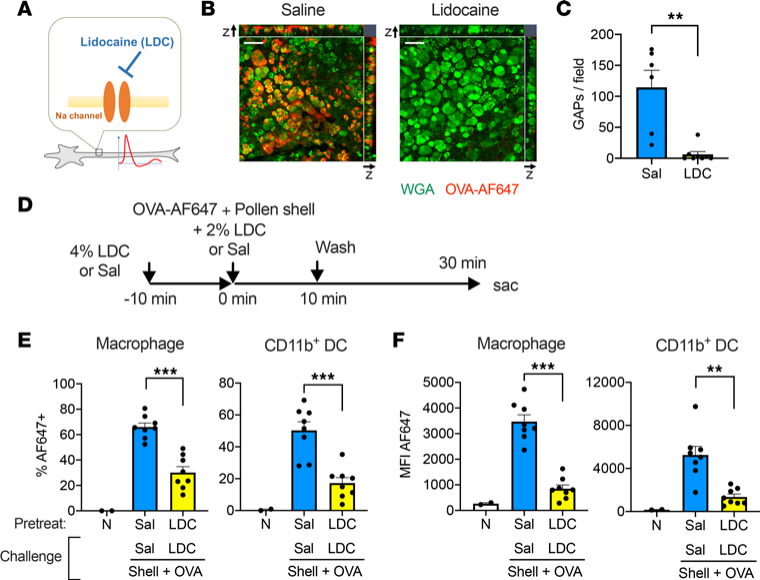
Topical lidocaine inhibits RW pollen shell–stimulated GAP formation and antigen uptake. (**A**) Lidocaine blocks Na^+^ channels in neurons. (**B** and **C**) GAP formation after instillation of OVA-AF647 and RW pollen shells. The indicated formula was also instilled 10 minutes prior to and together with OVA-AF647. Representative image (**B**) and quantitation (**C**) (*n* = 6–8). Sal, saline. Scale bars: 50 μm. **P < 0.01 by 2-tailed Mann-Whitney test. (**D**) The diagram of the antigen passage experiment. LDC, lidocaine. (**E** and **F**) The frequencies of OVA-AF647^+^ cells (**E**) and the mean fluorescence intensity (MFI) (**F**) of the indicated cell populations (*n* = 2–8). N, nontreated. Data are shown as mean ± SEM (**C**, **E**, and **F**). The nontreated samples were used for setting the positive gate and were excluded from the statistical analysis. **P < 0.01, ***P < 0.001 by 2-tailed Mann-Whitney test. B6 mice were used for all experiments.

The nervous system may respond rapidly to mechanical stimuli. To assess the nervous system involvement, a local anesthetic, lidocaine, was used to inhibit the axonal electrical transmission (Figure 5A). Although lidocaine is an acidic substance, the pH of our lidocaine formulation was between 6 and 7, which would not affect GAP formation (Supplemental Figure 7). Interestingly, topical lidocaine treatment almost completely abrogated the GAP formation (Figure 5, B and C). In addition, antigen uptake via the early antigen passage was markedly reduced (Figure 5, D–F). These results indicate that most of the early antigen passage was due to the GAP formation regulated by the nervous system. However, the remaining small amount of antigen transport in the absence of GAPs also suggest that the mechanistic disruption of the epithelial barrier provoked by the pollen granules and/or other modes of transepithelial transport may also be involved.

### mAchRs are not essential for GAPs and early antigen passage.

**Figure 6:**
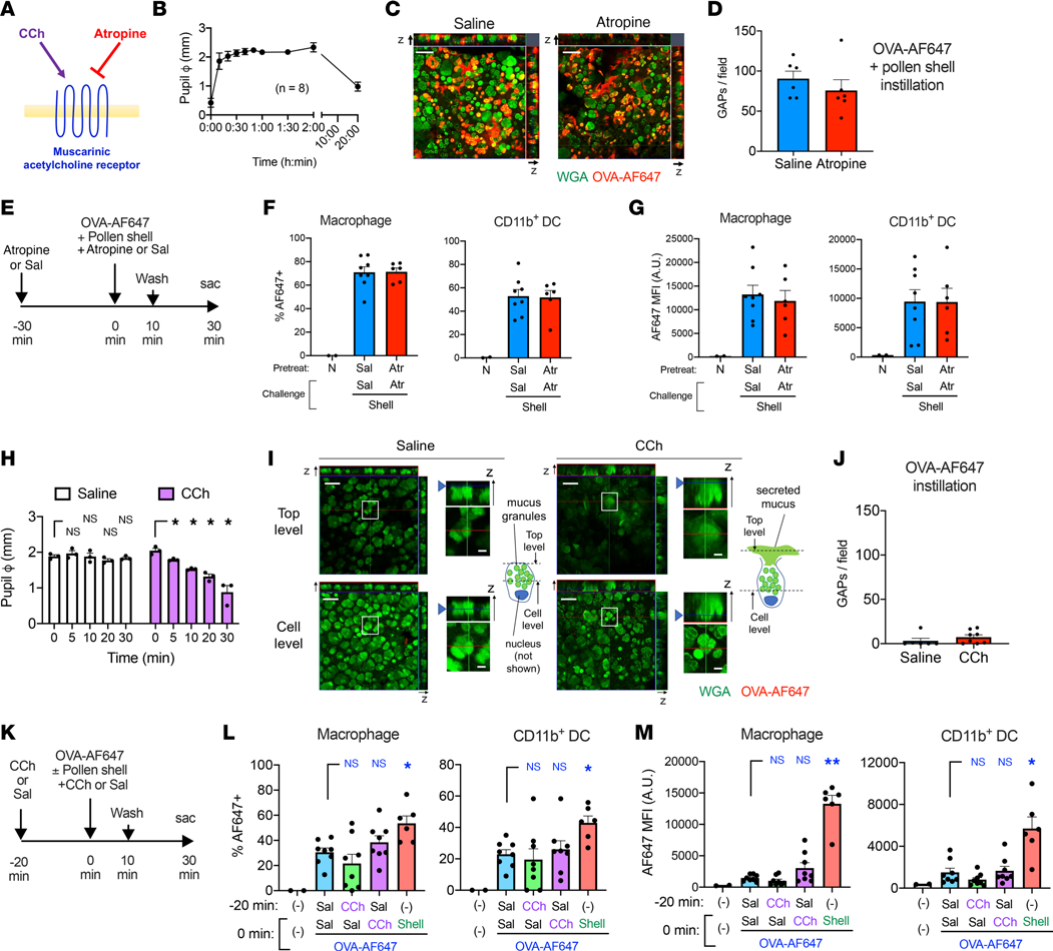
Muscarinic acetylcholine receptors (mAchRs) are not essential for RW pollen shell–stimulated GAP formation. (**A**) mAchRs are stimulated by carbamylcholine (CCh) and inhibited by atropine (Atr). (**B**) Kinetics of mydriasis after atropine instillation. (**C** and **D**) GAP formation 5 minutes after instillation of OVA-AF647 and pollen shells. The indicated formula was instilled 30 minutes prior to the challenge. Representative image (**C**) and quantification (**D**) (*n* = 6, each). Scale bar: 50 μm. (**E**) Diagram of the antigen passage experiment. Sal, saline. (**F** and **G**) The frequencies of OVA-AF647^+^ cells (**F**) and the mean fluorescence intensity (MFI) (**G**) of the indicated cell types (*n* = 2–8). N, nontreated. (**H**) Kinetics of miosis after CCh instillation to the euthanized mice (*n* = 3, each). *P < 0.05 by 2-way ANOVA with Holm-Šidák multiple-comparison test. (**I** and **J**) GAP formation and mucus secretion after instillation of OVA-AF647 along with the indicated formula. Representative images (**I**) and GAP quantitation (**J**) (*n* = 6–8). Scale bars: 50 μm (lower magnification) and 10 μm (insets). Annotated diagrams of the goblet cells in the insets are also shown. (**K**) Diagram for the antigen passage experiment in **L** and **M**. (**L** and **M**) The frequencies of OVA-AF647^+^ cells (**L**) and the MFI (**M**) of the indicated cell types. The nontreated samples were used for setting the positive gate and were excluded from the statistical analysis. *P < 0.05, **P < 0.01 by 1-way ANOVA with Holm-Šidák multiple-comparison test (**L**) and Kruskal-Wallis test with Dunn’s multiple-comparison test (**M**) against saline- and OVA-AF647–treated mice (*n* = 2–8). In **L** and **M**, (–) indicates no treatment. Data are shown as mean ± SEM (**B**, **D**, **F**–**H**, **J**, **L**, and **M**). B6 mice were used for all the experiments.

We further attempted to elucidate which nervous system regulates GAP formation. Conjunctival goblet cells express mAchRs and release mucus granules upon acetylcholine stimulation (9, 37, 38). Blockade of mAchR inhibits the formation of GAPs in intestinal goblet cells (35). Hence, we examined whether GAPs could be inhibited by atropine, a pan-mAchR antagonist, at a dose that can induce long-lasting mydriasis (Figure 6, A and B). However, the number of GAPs was only marginally reduced, without statistical significance, in the presence (Figure 6, C and D) or absence of pollen shells (Supplemental Figure 8). Furthermore, antigen uptake levels were not altered (Figure 6, E–G). Conversely, carbamylcholine (CCh), a cholinergic agent, was used to test whether mAchR stimulation can induce GAP formation. CCh at a functional dose that induces miosis (Figure 6H) and mucus secretion (Figure 6I) tended to increase the number of GAPs (Figure 6J), although the number of GAPs was still very small compared with that of GAPs induced by pollen shells (Figure 6, C, D, I, and J). Antigen uptake showed a similar, but statistically insignificant tendency (Figure 6, K–M). These results suggest that mAchR is not a major trigger for conjunctival GAP formation, unlike intestinal GAPs.

### The trigeminal nerves are involved in GAP formation.

**Figure 7:**
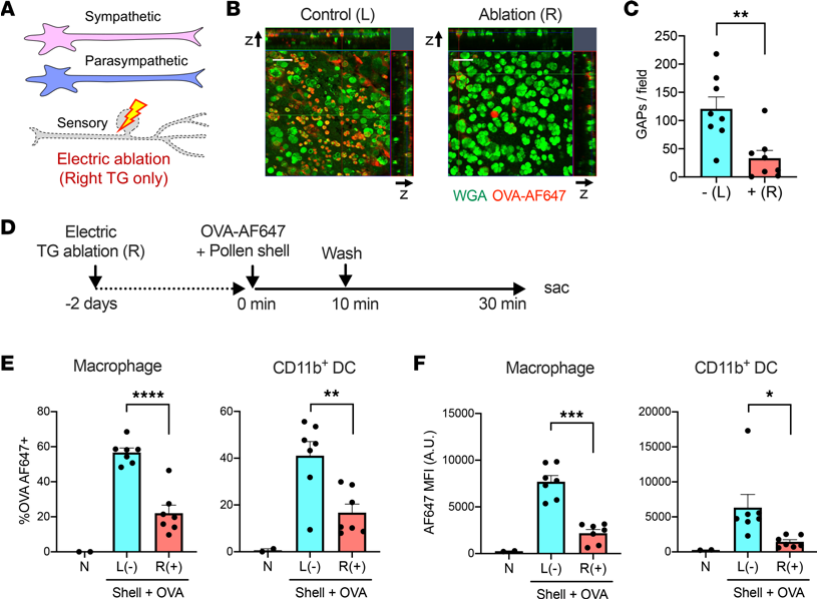
Trigeminal nerve ablation inhibits RW pollen shell–stimulated GAP formation and early antigen uptake. (**A**) The trigeminal (TG) nerve was ablated by bipolar coagulation. (**B** and **C**) GAP formation 5 minutes after instillation of OVA-AF647 and pollen shells. Representative image (**B**) and quantitation (**C**) (*n* = 8). Scale bars: 50 μm. **P < 0.01 by 2-tailed, paired Student’s *t* test. (**D**) Experimental diagram of the early antigen passage after TG ablation. (**E** and **F**) The frequencies of OVA-AF647^+^ cells (**E**) and the mean fluorescence intensity (MFI) (**F**) of the indicated cell types (*n* = 2–7). The nontreated samples were used for setting the positive gate and were excluded from the statistical analysis. In **C**, **E**, and **F**, – or (–) indicates no TG ablation and + or (+) indicates TG ablation. *P < 0.05, **P < 0.01, ***P < 0.001, ****P < 0.0001 by 2-tailed, paired Student’s *t* test. B6 mice were used for all experiments.

Finally, we investigated whether sensory nerves are required for sensing particulate pollens and/or for GAP formation. Electrocoagulation was performed to ablate the first branch of the trigeminal (TG) nerve (39). The TG nerve of 1 side was ablated, leaving the other side as the sham-operated control (Figure 7A). Intriguingly, GAP formation was almost completely abolished on the TG-ablated side (Figure 7, B and C). Furthermore, early antigen passage was also markedly reduced on the ablated side (Figure 7, D–F). These results indicate that the sensory nervous system may be required to sense particulate antigens and/or to trigger GAP formation.

## Discussion

The entry of allergens into conjunctival tissue is the initial step in the development of allergic conjunctival diseases. Several pathways mediating the acquisition of antigens through the conjunctiva have been proposed, including defects in the glycocalyx (40), defects in the intercellular junctions (41, 42), transepithelial dendrites of DCs (43), and GAP formation (7, 9, 44). The present study showed that OVA, which essentially lacks protease activity, can enter the conjunctiva, and cause eosinophilic inflammation when administered along with pollen shells. Pollen shells indirectly stimulate goblet cells through the nervous system to form GAPs, which facilitate antigen uptake by CD11b^+^ DCs and macrophages. Early antigen passage was sufficient for the development of eosinophilic conjunctivitis, suggesting that a large allergen influx is caused by GAP formation. Inhibition of intrinsic mechanical sensory mechanisms by Gd(III) (34) did not inhibit the GAP formation or antigen uptake. In addition, pollen shell–stimulated IL-33 release did not play a role. Contrary to expectations, the a pan-mAchR antagonist atropine only marginally reduced the GAP formation and antigen uptake. Conversely, the acetylcholine receptor agonist did not induce a significant increase in the number of GAPs or antigen passage. Because the use of lidocaine and electric ablation of the TG nerve had a suppressive effect on GAP formation and early antigen passage, neurotransmitters other than acetylcholine may be used to ignite the GAP formation in the pollen shell–stimulated conjunctiva. Our results indicate that the regulatory mechanisms underlying the rapid GAP formation in the conjunctiva are distinct from those regulating the GAPs in the intestine.

The conjunctiva receives parasympathetic, sympathetic, and sensory innervation from the pterygopalatine, superior cervical, and TG ganglia, respectively (45, 46). Among them, the nerve endings of the parasympathetic and sympathetic nerves were reported to colocalize with goblet cells, while those of sensory nerves was detected only in the epithelium and at the epithelial stromal junction (47). Consistently with this, the human and mouse goblet cells express both muscarinic and adrenergic receptors (48). Furthermore, goblet cells secrete mucus when stimulated by parasympathetic neurotransmitters, including acetylcholine and vasoactive intestinal peptide (37, 38, 49). In the intestine, GAP formation is stimulated by acetylcholine but not by cholera toxin, although both can stimulate mucus release from goblet cells (35). Therefore, not all secretagogues contribute to the formation of GAPs. Our results indicate that the major parasympathetic neurotransmitter, acetylcholine, may not play a significant role in driving conjunctival GAP formation with or without the pollen shell stimuli. In addition, various degrees of mucus secretion were observed in GAP-forming goblet cells, suggesting that compound exocytosis that empties goblet cells (50, 51) is not a prerequisite for GAP formation. In order to identify the responsible nerves and transmitters, further denervation studies, or studies using various receptor antagonists acting on neurotransmitter receptors are warranted.

In conjunctival provocation tests (CPTs), reconstituted lyophilized allergen extract is used as a standardized allergen without the addition of pollen shells. Clinical signs and symptoms, including itchiness, usually start to appear within several minutes and peak at around 10–20 minutes (52). This indicates that soluble antigen alone can penetrate into the conjunctiva, and induce allergic responses in some of the sensitized patients. In our animal models, a small amount of antigen uptake by antigen-presenting cells was observed, even without the presence of pollen shells. However, as shown in this study and in our previous study (31), soluble antigens alone failed to elicit eosinophilic conjunctivitis. As defects in intercellular junctions have been observed even out of season in SAC patients (41), the permeability of the conjunctiva might be increased in the SAC patients after several rounds of seasons. In contrast, animal models start with a normal conjunctiva. This difference may in part explain why pollen shell–induced GAPs are required in animal models. Conversely, early SAC patients may not properly respond to CPT in the absence of pollen shells, if performed out of season. In line with this, Radcliffe et al. reported that preseasonal CPT results had no correlation with the eye domain quality of life score during the peak week of the corresponding pollen season, even though their CPT results were highly reproducible (53). Kruse et al. examined the differences in seasonal symptom scores between patients with positive preseasonal CPT results and those with negative results after sublingual immunotherapy (54). The study involved 2 sets of trials: a grass pollen study and a tree pollen study. Although CPT-positive and CPT-negative populations showed a significant difference in total clinical scores, only the grass pollen study showed a statistically significant difference in ocular symptom scores, with a substantial overlap between the groups (54). These observations suggest that preseasonal CPT has limitations in predicting seasonal ocular symptoms. Since shell preparation can be industrially standardized (55), the combinations of standardized pollen shells and standardized extracts may improve the accuracy of CPT in predicting the severity of seasonal symptoms.

In summary, we have identified pollen shell–induced massive formation of conjunctival GAPs that are formed through different mechanisms than those for intestinal GAPs and a previously unappreciated role of GAPs in the development of allergic conjunctivitis. Further studies are warranted to elucidate the specific neurotransmitters and pathways responsible for conjunctival GAP formation and development of allergic conjunctival diseases.

## Methods

### Mice.

B6 and BALB/c mice were bred in the animal facility of Juntendo University or purchased from Sankyo Labo Service Corporation. ST2-knockout mice have been described previously (31). FcεRIα-knockout mice were generated from B6.Cg-*Fcer1^atm1Knt^* Tg(FCER1A)1Bhk/J (Jackson Laboratory) by backcrossing to B6 mice to remove the human FcεRIα transgene.

### Labeling OVA with AF647-succinimidyl ester.

OVA (A5503, Merck) was labeled with AF647-NHS ester (A20006, Thermo Fisher Scientific) following the manufacturer’s instructions. Briefly, OVA was dissolved at a concentration of 20 mg/mL in PBS and the pH was adjusted by adding 1/10 volume of 1 M NaHCO_3_ (pH 8.3). After centrifugation at 20,000*g* for 10 minutes, the supernatant was collected. The labeling reagent was prepared by dissolving 1 mg of AF647-NHS ester in 100 μL of DMSO and was added to the OVA solution. The mixture was incubated at room temperature for 60 minutes with continuous stirring. After centrifugation, the unbound dye was removed by size fractionation using PD-10 columns (Cytiva). Size fractionation was performed twice. OVA-AF647 was concentrated using Amicon Ultra 4 Centrifugal Filters (MWCO 3 kDa, Merck), and the filtrate appeared colorless. The product was filtered through a 0.22-μm filter using a Costar Spin-X column (Corning) and stored at 4°C until use.

### Pollen shell preparation.

RW pollen shells were prepared as previously described (31, 55). Briefly, the RW pollens were defatted with acetone under reflux at 60°C overnight. The shell content was removed by incubation in phosphoric acid at 60°C for 7 days. After serial washings with hot water, acetone, 2 M HCl, 2 M NaOH, water, acetone, and ethanol, the shells were incubated in a 6% w/v KOH solution at 80°C for 6 hours. After centrifugation, the KOH treatment was repeated. The pollen shells were serially rinsed with hot water and acetone, and then dried overnight. The pollen shells were weighed and suspended in PBS to determine particle concentrations. The residual protein content was measured using a Pierce BCA Protein Assay Kit (Thermo Fisher Scientific) following the manufacturer’s instructions, and was confirmed to be less than 5% of the original RW preparations.

### Allergic conjunctivitis models.

The local sensitization model of allergic conjunctivitis was induced in B6 mice, as described previously (31). Briefly, the mice received repeated topical applications of pollen suspension (0.5 mg in 2.5 μL of PBS) 5 days a week for 3 weeks without prior sensitization. Two days later, the last challenge was applied. The mice were sacrificed 24 hours after the last challenge and analyzed. In some experiments, mice received 40 mg/mL OVA dissolved in PBS, and/or RW pollen shells at the same particle concentrations as that of the 200 mg/mL pollen suspension.

The systemically sensitized model of allergic conjunctivitis was induced in BALB/c mice following a previously published protocol, with slight modifications (56, 57). Briefly, each foot pad of BALB/c mice was injected with 50 μg of RW pollen (*Ambrosia artemisiifolia*, from Polysciences) emulsified in 50 μL of ImjectAlum Adjuvant (Thermo Fisher Scientific) on day 0, followed by an intraperitoneal injection of 0.1 mg of RW pollen suspension in 200 μL of PBS on day 14. Each eye was challenged with 2.5 μL of 200 mg/mL RW pollen suspension in saline or vehicle once a day from day 28 to 31. In some experiments, the mice received eye washes with approximately 15 mL of PBS after the indicated periods. The mice were sacrificed 24 hours after the last challenge, and the harvested tissues were analyzed.

### Ablation of TG nerve.

The stereotactic coagulation of the first branch of the intracranial TG nerve has been described previously (39). Briefly, a small skin incision was made to expose the cranium under general anesthesia. A small hole was created in the cranium above the first branch of the right TG nerve using an 18-G needle. A bipolar tip (Wet-Field Hemostatic Eraser Bipolar 20G, 221260, BVI Medical) was inserted into the brain at a depth of 6 mm. The nerve was coagulated for 3 minutes using a microsurgical bipolar coagulator (TI-B40ac, Natsume Seisakusho) set at 500 Ω and 10 W. Sensory loss was confirmed by the lack of the blink reflex upon air jet stimulation.

### Evaluation of GAPs using tissue clearing.

The mice were euthanized 5 minutes after OVA-AF647 and/or shell administration, and the conjunctival tissue was immediately fixed with 4% paraformaldehyde (PFA) (Fujifilm) in situ for 5 minutes. The entire conjunctiva was dissected and further fixed in 4% PFA for 3 days at 4°C. After fixation, the tissue was subjected to the AbScale tissue clearing protocol described previously (33). Briefly, the fixed conjunctiva was incubated in SCALEVIEW-S0 (Fujifilm) for 4 hours at 37°C. Samples were then immersed in SCALEVIEW-A2 (Fujifilm) for 8 hours, followed by Scale B4 (8 M urea) for 12 hours and SCALEVIEW-A2 for 8 hours at 37°C. The samples were restored using deScale Solution for 6 hours at 4°C. Then, the samples were stained with CF488A-labeled WGA (Biotium) in AbScale solution (0.33 M urea, 0.1% Triton X-100 in 1× PBS) for 24 hours at 37°C. After washing with AbScale solution twice (first 2 hours, second 1 hour) at room temperature, the samples were refixed in 4% PFA at room temperature for 1 hour. Finally, the conjunctiva was incubated in SCALEVIEW-S4 (Fujifilm) for 6–8 hours at 37°C for clearing. Incisions were made to flatten the tissue, and a coverslip was mounted using SCALEVIEW-S4. The fluorescence was imaged using an LSM 700 confocal microscope (Carl Zeiss). The GAPs in the upper and lower conjunctiva were counted in the 0.1-mm^2^ fields that contained the largest number of GAPs. The average number of GAPs in the upper and lower conjunctivae was calculated. The fluorescence intensity was calculated from the maximal intensity projection of the conjunctival surface layers and the stromal layers of the same area using Image J software (version 1.53, NIH).

### Flow cytometry.

The mouse conjunctiva was harvested after euthanasia and cut into small pieces using scissors. The tissue was enzymatically digested in RPMI 1640 medium (Fujifilm) supplemented with 10% FCS, 2 mg/mL collagenase (Fujifilm), and 0.1 mg/mL DNase (Roche Diagnostics GmbH) for 1 hour at 37°C with continuous stirring. The digested tissue was filtered through a nylon mesh. A part of the filtered cell suspension (20 μL) was collected for cell counting. The remainder was centrifuged at 300*g* for 5 minutes at 4°C, and the supernatant was aspirated. The cell pellet was resuspended in FACS buffer (2% FCS and 0.1% NaN_3_ in PBS). After washing, the cells were blocked with anti-CD16/anti-CD32 antibody (clone 2.4G2) for 10 minutes at 4°C and further stained with surface-staining antibodies for 20 minutes at 4°C. The following antibodies were used: Anti–Siglec-F AF647 (clone E50-2440, catalog 562680) and BV711 Streptavidin (catalog 563262) were from BD Biosciences. Anti-CD11b FITC (clone M1/70, catalog 101206), anti-CD11c PE/Cyanine 7 (clone N418, catalog 117318), anti-CD16/32 PE (clone 93, catalog 101308), anti-CD326 (Ep-CAM) Alexa Fluor 488 (clone G8.8, catalog 118210), anti-CD3ε PE/Cyanine 7 (clone 145-2C11, catalog 100320), anti-CD4 APC/Fire 750 (clone GK1.5, catalog 100460), anti-CD45 Biotin (clone 30-F11, catalog 103104), anti-CD45 PerCP/Cyanine 5.5 (clone 30-F11, catalog 103132), anti-CD64 PE/Cyanine 7 (clone X54-5/7.1, catalog 139314), anti-CD80 PE (clone 16-10A1, catalog 104708), anti-CD86 PE (clone GL-1, catalog 105008), anti-FcεRIα FITC (clone MAR-1, catalog 134306), anti–I-A/I-E PerCP/Cyanine 5 (clone M5/114.15.2, catalog 107614), anti-IgE Alexa Fluor 488 (clone RME-1, catalog 406910), and anti-Ly-6G APC/Fire 750 (clone 1A8, catalog 127652) were from BioLegend. Anti-CD117 (c-Kit) APC (clone 2B8, catalog 17-1171-83) was from Thermo Fisher Scientific. Anti-T1/ST2 PE (clone DJ8, catalog 101001PE) was from MD Bioproducts. Anti-CCR3 PE (clone 83101, catalog FAB729P) was from R&D Systems. Anti-CD11c PE (clone N418, catalog 50-0114-U100) was from TONBO Biosciences. Macrophages and CD11b^+^ DCs were identified as CD11c^–^MHC II^+^CD11b^+^ cells and CD11c^+^MHC II^+^CD11b^+^ cells, respectively, in the DAPI^–^CD45^+^ single-cell populations. Flow cytometry was performed using a FACSVerse (BD Biosciences). Data were analyzed using FlowJo software (v.10.6.2, BD Biosciences).

### Histology.

Mouse conjunctival biopsies were fixed in 4% PFA, and Giemsa staining was performed on the paraffin-embedded sections.

For the cryosections, the mouse conjunctiva was fixed in situ with 10 μL of 4% PFA for 5 minutes after euthanasia. Thereafter, the conjunctiva was collected and further fixed for 25 minutes in total. The tissue was rinsed thoroughly with PBS, embedded in OCT compound (Sakura Finetek), and frozen. The 6-μm cryosections were stained with DAPI (Dojindo) and coverslips were mounted with SlowFade Diamond Antifade Mountant (Thermo Fisher Scientific). The sections were imaged using an LSM 700 confocal microscope system.

### Antigen passage through the conjunctiva and antigen uptake by immune cells.

Each mouse conjunctival sac was instilled with 2.5 μL of OVA-AF647 and/or RW pollen shells that are of the same particle concentration as that of the 200 mg/mL RW pollen suspension. In some experiments, 50 μM Gd(III)Cl_3_ (FUJIFILM), 100 μM CCh (FUJIFILM), 1% atropine (Nitten Pharmaceutical), 4% or 2% lidocaine (Aspen Japan), 0.1% epinastine (Santen Pharmaceutical), PBS pH 6, 7, or 8, or saline was applied to the eyes prior to and/or together with OVA-AF647 as indicated. To assess the early antigen passage through the conjunctiva, the eyes were washed with PBS pH 7.4 in some experiments. In these cases, the mice were sacrificed 30 minutes after the instillation of OVA-AF647 to allow immune cells to acquire antigens.

### Statistics.

Statistical analysis was performed using Prism 8 software (GraphPad). Data are presented as mean ± SEM in all figure parts in which error bars are shown. The differences between groups were analyzed using a 2-tailed Student’s *t* test with Welch’s correction, Mann-Whitney test, 1-way or 2-way ANOVA with Holm-Šidák multiple-comparison test, or Kruskal-Wallis test with Dunn’s multiple-comparison test, as indicated. A *P* value of less than 0.05 was considered statistically significant.

### Study approval.

All animal experiments were approved by the ethical committee of Juntendo University (approval number 2020136) and performed in accordance with the Association for Research in Vision and Ophthalmology (ARVO) statement for the animal use in ophthalmic and vision research (https://www.arvo.org/About/policies/arvo-statement-for-the-use-of-animals-in-ophthalmic-and-vision-research/).

### Data availability.

All data presented in the manuscript and supplemental material are provided in the Supporting Data Values file.

## Author contributions

TA and JK conceived the project. MK and TA designed the study. MK, TA, YK, SF, MM, KK, KI, and AK performed experiments. MK and TA analyzed and interpreted the results and prepared figures. NN, KM, HO, KO, SN, AM, NE, and JK contributed to the interpretation of data. MK and TA drafted the manuscript. JK and KO supervised the entire project. All authors provided critical feedback and helped shape the research, analysis, and manuscript.

## Supplementary Material

Supplemental data

Supporting data values

## Version 1 10/11/2023

In-Press Preview

## Version 2 11/08/2023

Electronic publication
