# Supplementary material for: A nerve-goblet cell association promotes allergic conjunctivitis through rapid antigen passage
Source: JCI Insight. 2023 Nov 8;8(21):e168596. doi: 10.1172/jci.insight.168596 (PMC10721269; doi:10.1172/jci.insight.168596)
Supplement: Supplemental data [file jciinsight-8-168596-s169.pdf]

Supplemental Material

A nerve-goblet cell association promotes allergic conjunctivitis through the rapid antigen passage

Meiko Kimura, Tomoaki Ando, Yasuharu Kume, Saaya Fukase, Moe Matsuzawa, Kosuke Kashiwagi, Kumi Izawa, Ayako Kaitani, Nobuhiro Nakano, Keiko Maeda, Hideoki Ogawa, Ko Okumura, Shintaro Nakao, Akira Murakami, Nobuyuki Ebihara, and Jiro Kitaura

Content:

Supplemental Figures 1-8

Supplemental Figures

Supplemental Figure 1

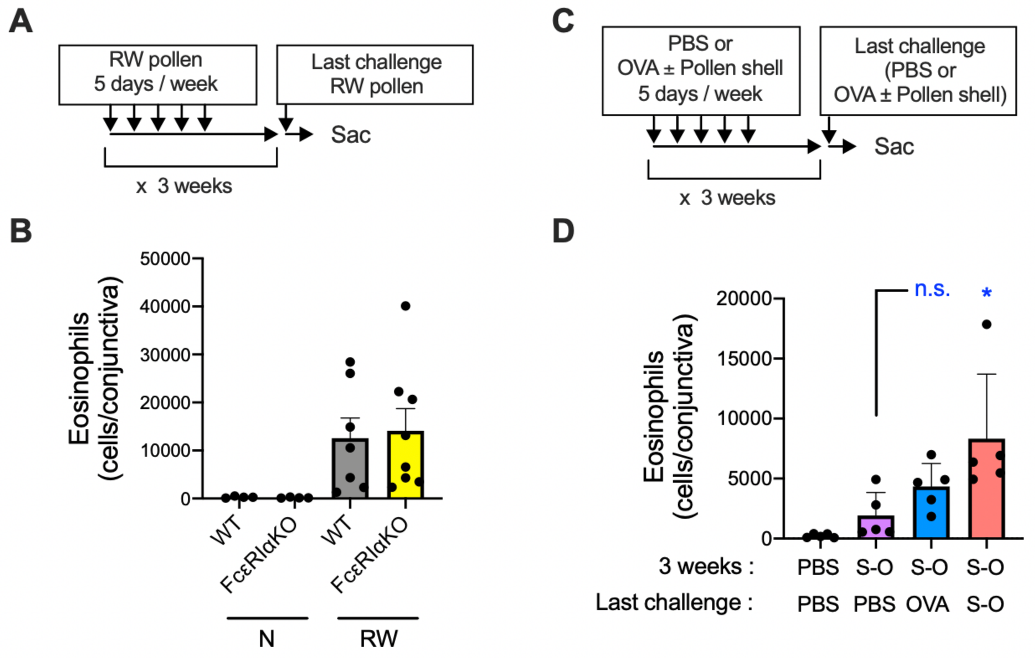

Characterization of the local sensitization model of allergic conjunctivitis. (A) The diagram for the local sensitization model. RW, ragweed. Sac, sacrifice. (B) Eosinophils were enumerated in the conjunctiva using FACS. N, non-treated mice. (C) The experimental diagram for testing the effect of pollen shells in the last challenge. (D) Eosinophils were enumerated in the conjunctiva treated with the indicated formula. S-O, pollen shell and ovalbumin. **p*<0.05 by two-tailed one-way ANOVA with Holm-Sidak's multiple comparisons against S-O sensitized PBS challenged condition. Non-sensitized (PBS) PBS challenged condition was excluded from the statistical analysis. C57BL/6 (B6) background mice were used in all the experiments.

Supplemental Figure 2

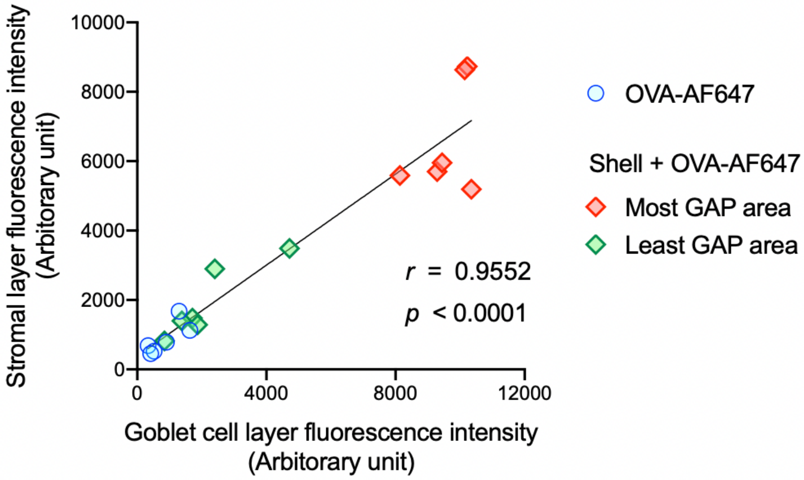

Antigen fluorescence intensities of conjunctival surface correlate with those of the stroma. The fluorescence intensities of OVA-AF647 were measured in the surface goblet cell layers and in the stromal layers of the conjunctiva instilled with OVA-AF647 or pollen shell and OVA-AF647. Both the areas with the most GAPs and the least GAPs were measured in the pollen shell and OVA-AF647-instilled eyes. Pearson’s correlation coefficient and a p-value is shown. B6 mice were used.

Supplemental Figure 3

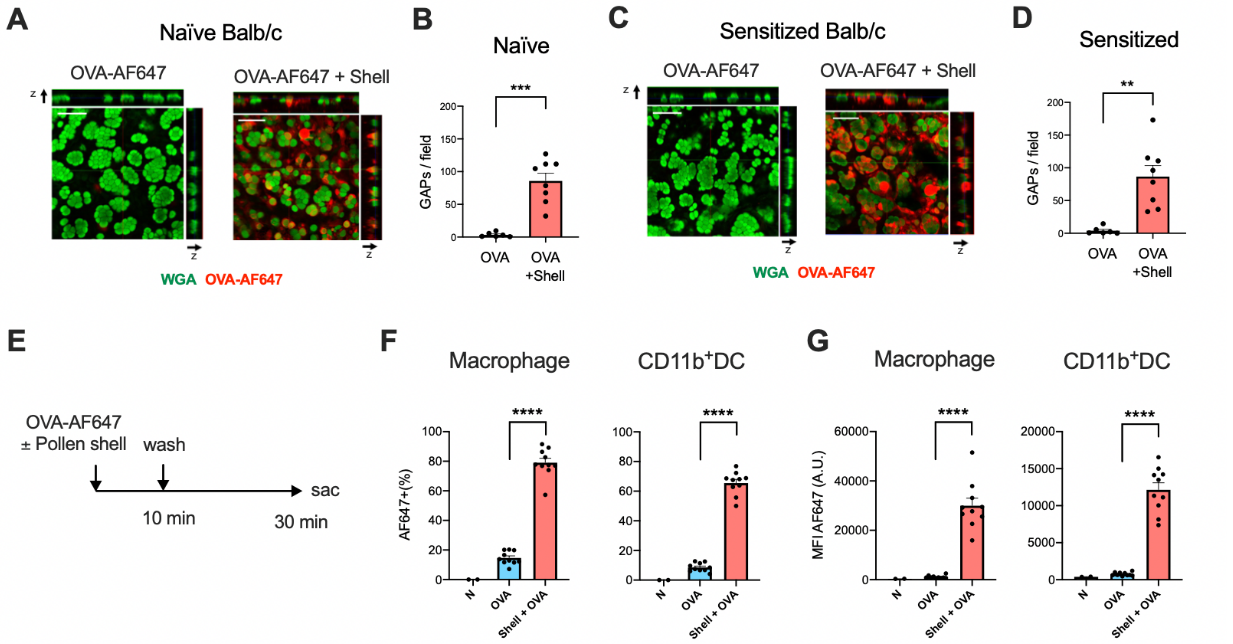

Pollen shells promote GAP formation and the antigen uptake in the Balb/c conjunctiva regardless of the sensitization status. (A-D) Naïve or systemically sensitized Balb/c mice were challenged with OVA-AF647 and/or pollen shells. Representative images (A,C) and enumeration of GAPs (B,D) are shown. Bar, 50 μm. $**p<0.01$, $***p<0.001$ by two-tailed t-test with Welch's correction. (E) The experimental diagram for the OVA-AF647 uptake experiment. (F,G) Frequencies of OVA-AF647+ cells and mean fluorescence intensity (MFI) in the indicated cell populations. A.U., arbitrary unit; N, non-treated. The non-treated samples were used for setting the positive gate purpose and were excluded from the statistical analysis. $****p < 0.0001$ by two-tailed t-test with Welch's correction. Balb/c mice were used in all the experiments.

Supplemental Figure 4

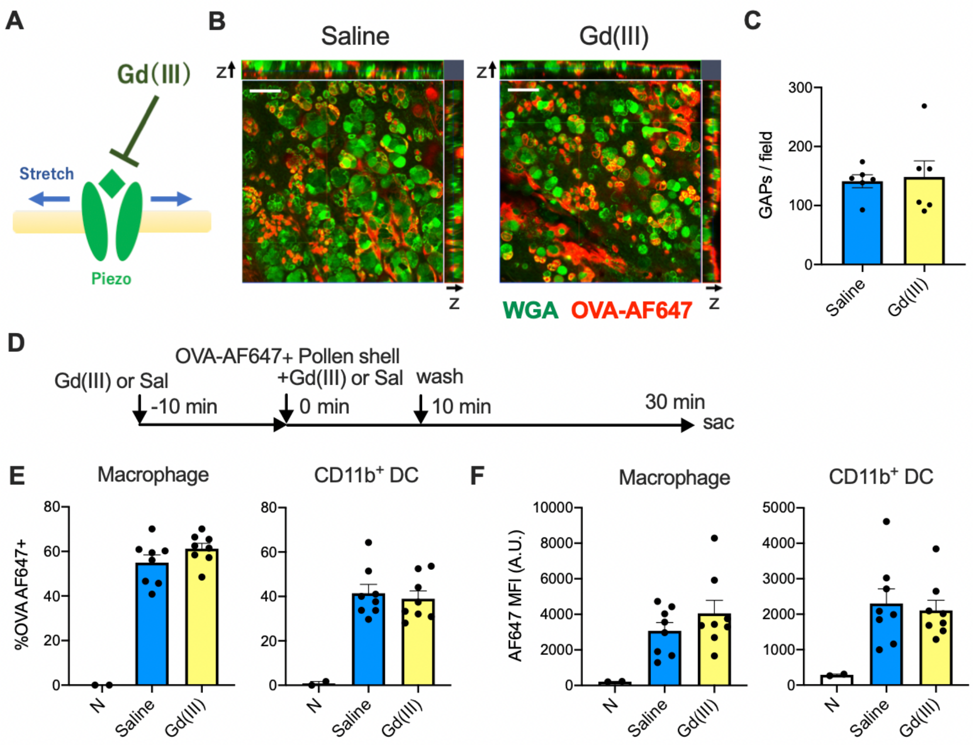

Piezo1 is not responsible for GAP formation and the early antigen uptake. (A) Gd(III) chloride inhibits Piezo1 and Piezo2 mechanoreceptors. (B,C) GAP formation at 5 min after instillation of OVA-AF647 and pollen shells. Indicated formula was also instilled at 10 min prior to and together with OVA-AF647. Representative image (B) and quantification (C). Bar, 50 μm. (D) The diagram for the antigen passage experiment. Sal, saline; sac, sacrifice. (E,F) The percentage (E) and the amount (F) of the antigen uptake by indicated cell types in the conjunctiva. N, non-treated. Non-treated samples were prepared for gating purpose only, therefore were excluded from the statistical comparison. The differences of the means were not significant by two-tailed Student's t-tests with Welch's correction. B6 mice were used in all the experiments.

Supplemental Figure 5

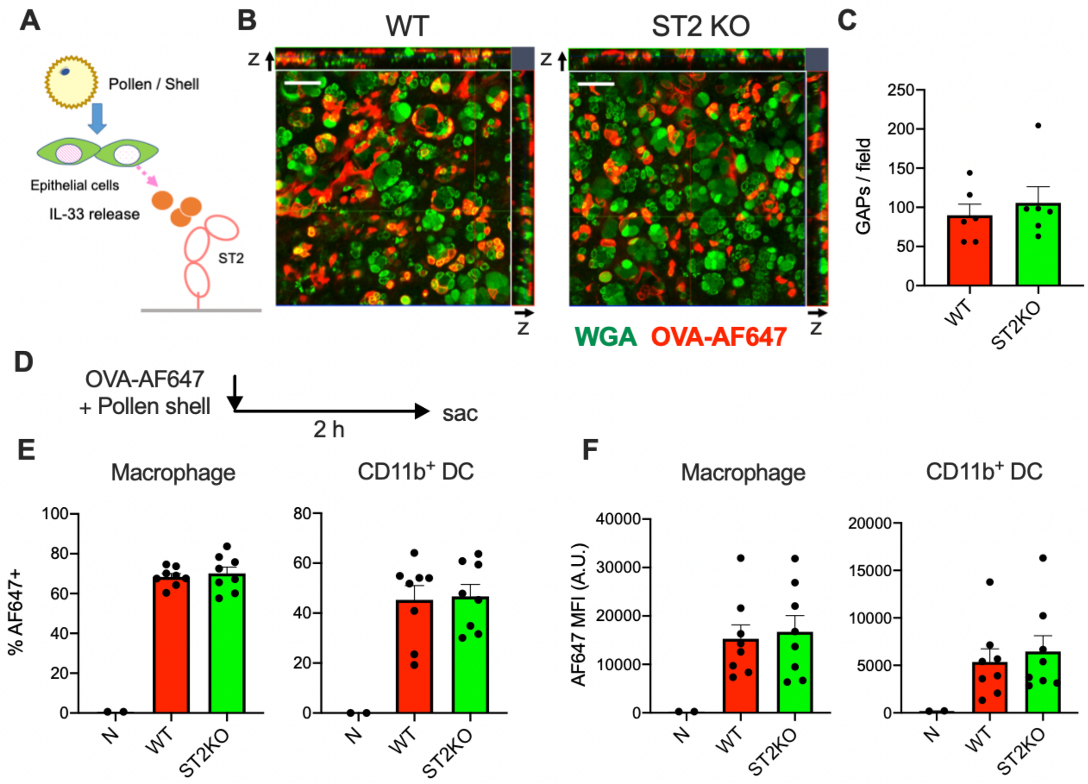

IL-33 receptor, ST2, is not responsible for GAP formation and the early antigen uptake. (A) Pollen shells stimulate the epithelial cells directly or indirectly to release IL-33. Released IL-33 acts on its receptor ST2 on the cell surface. (B,C) GAP formation at 5 min after instillation of OVA-AF647 and pollen shells. KO, knockout. Representative image (B) and quantitation (C). Bar, 50 µm. (D) The diagram for the antigen passage experiment. sac, sacrifice. (E,F) The percentage (E) and the amount (F) of the antigen uptake by indicated cell types in the conjunctiva. N, non-treated wild type. Non-treated samples were prepared for gating purpose only, therefore were excluded from the statistical comparison. The differences of the means were not significant by two-tailed Student's t-tests with Welch's correction. B6 background mice were used in all the experiments.

Supplemental Figure 6

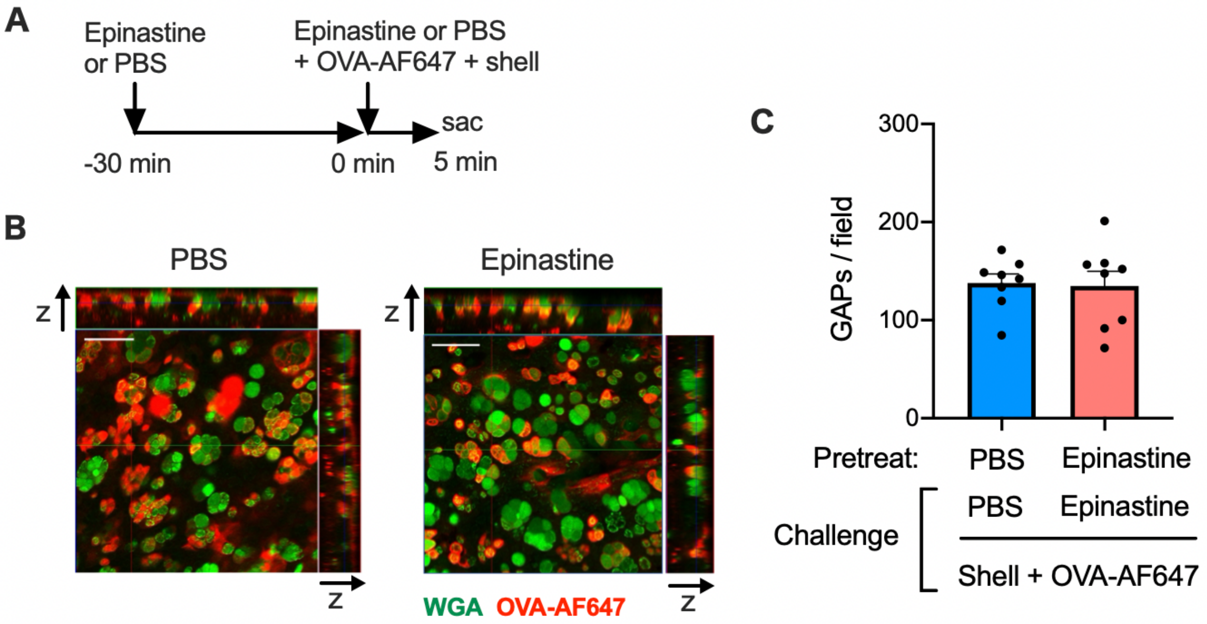

A histamine H1 receptor inverse agonist does not affect GAP formation. (A) The experimental diagram. (B,C) GAP formation at 5 min after instillation of OVA-AF647 and pollen shells, with or without epinastine pretreatment. Representative images (B) and quantification (C). Bar, 50 μm. B6 background mice were used.

Supplemental Figure 7

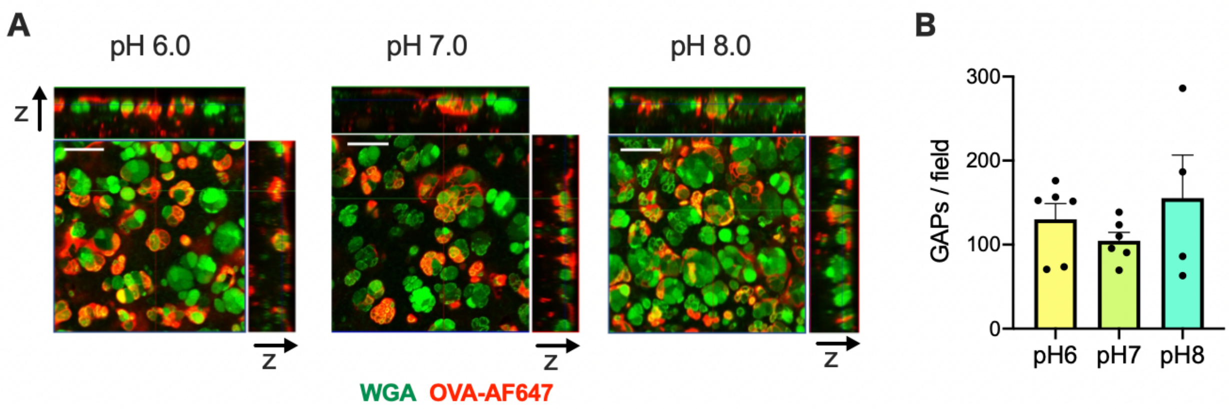

Close to neutral pH does not affect GAP formation. (A,B) GAP formation at 5 min after instillation of OVA-AF647 and pollen shells, formulated in phosphate buffers of indicated pH. Representative images (A) and quantification (B). Bar, 50 µm. B6 background mice were used.

Supplemental Figure 8

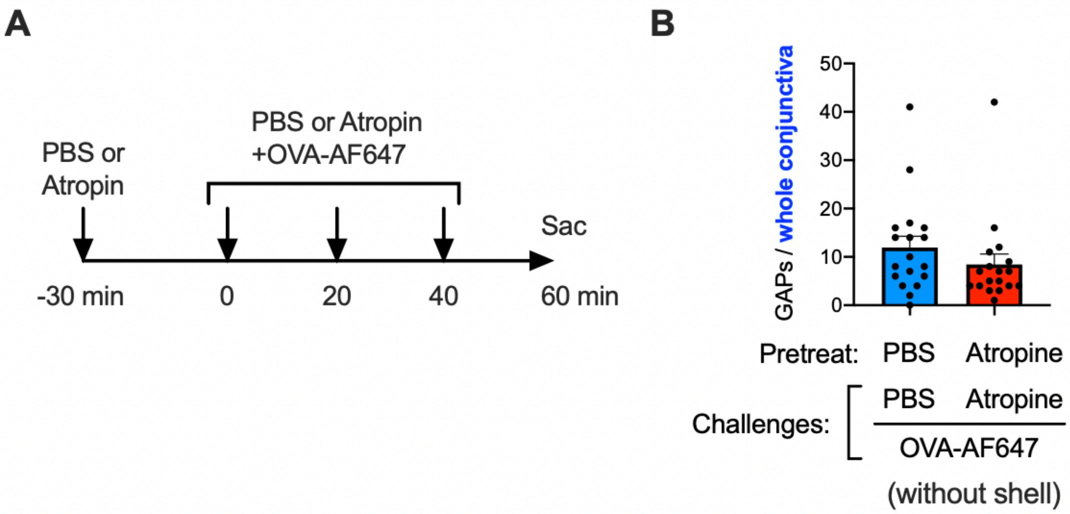

Background levels of GAP formation were not affected by the atropine treatment. (A,B) The experimental diagram (A) and the GAP enumeration in the whole conjunctiva (B). Mouse eyes were treated with PBS or atropine 30 min before and during GAP detection. Accumulated GAPs during the three rounds of OVA-AF647 instillations (without pollen shells) were scanned for the whole conjunctiva and counted. A pooled result of two independent experiments. B6 background mice were used.
